# Long-term and low-dose of busulfan is a safe and effective second-line treatment in elderly patients with essential thrombocythemia resistant or intolerant to hydroxyurea

**DOI:** 10.1038/s41408-018-0091-6

**Published:** 2018-06-11

**Authors:** Rossella Renso, Andrea Aroldi, Pietro Pioltelli, Carlo Gambacorti-Passerini, Elena Maria Elli

**Affiliations:** 1 Hematology Division and Bone Marrow Unit, San Gerardo Hospital, Monza, Italy; 2 Department of Medicine, University of Milano-Bicocca, Monza, Italy; 3 Hematology Division and Clinical Research Unit, San Gerardo Hospital, Monza, Italy

Essential thrombocythemia (ET) is a Philadelphia negative myeloproliferative neoplasm (MPN) characterized by an increased risk of thrombosis and, in the long-term, transformation to myelofibrosis (MF) or acute leukemia^1^. The purpose of therapy is to achieve a good cytoreduction and to prevent thrombosis and bleeding complications without increasing the transformation risk. In patients at high risk of thrombosis, cytoreduction with hydroxyurea (HU) is currently recommended as first-line therapy^2^. Busulfan (BU) is an alkylating agent that has been used since 1959 for the treatment of MPN^3^. In some retrospective studies, its use according to the conventional schedule has been associated to high risk of leukemic transformation and second neoplasms, as with other alkylating agents. Consequently, BU is currently used by many hematologists as second-line treatment in Bcr-Abl negative MPN that are resistant to or developed side effects from HU, principally in elderly patients, for whom therapeutic options remain limited^4–6^. The conventional schedule provides a starting dose of 14 mg/week up to obtain a complete haematological response (CHR), defined according to the European LeukemiaNet (ELN) criteria ^7^.

In the current retrospective study, we wanted to share our experience with use of long-term and low-dose of BU in elderly patients with ET who are resistant or intolerant to HU.

From 1995 to 2015 we collected 348 patients who received ET diagnosis at our department, according to WHO criteria^1^. The principal clinical and hematological features of entire cohort of ET patients were shown in Table 1. In this population we identified 30 patients who received a second-line treatment with an alternative schedule of BU, defined by low-starting dose (4–6 mg/week) down to obtain CHR, followed by a dose de-escalation of BU over time up to the minimal dose required for the maintenance of CHR. The dose de-escalation of BU was performed every 4 weeks and the minimal dose to maintain the CHR was generally between 2 mg/week and 2 mg/month.

**Table 1:** Main clinical and hematological features of entire series of patients with essential thrombocythemia (ET) (*n* = 348) and of patients treated with (*n* = 26) or without (*n* = 322) Busulfan

Variables analyzed	Busulfan (*n* = 26)	No Busulfan (*n* = 322)	Entire cohort ET (*n* = 348)	*P*-value “Busulfan” vs. “no Busulfan”
Gender (male/female)	7/19	131/191	138/210	*P* = 0.17
Driver mutations (*n* (%)):				
JAK2V617F mutation	15 (57.7%)	201 (62.4%)	216 (62.1%)	*P* *=* 0.79
Calreticulin mutation	8 (30.8%)	60 (18.6%)	68 (19.5%)	*P* = 0.21
MPL mutation	1 (3.8%)	11 (3.4%)	12 (3.5%)	*P* = 0.91
Triple negative	2 (7.7%)	50 (15.6%)	52 (14.9%)	*P* = 0.28
Age (years) at diagnosis^a^	71.2 (52.7–85.2)	58.7 (12.1–92.1)	60.5 (12.1–92.1)	*P* < 0.001
Blood cell count at diagnosis^a^				
Hematocrit (%)	40.1 (28.6–50.4)	42 (29.3–55.5)	42 (28.6–55.5)	*P* = 0.028
Platelets count (×10^9^/L)	788.5 (507–2887)	674.5 (233–2240)	679.5 (233–2887)	*P* = 0.007
Leukocyte count (×10^9^/L)	8.3 (4.84–25.49)	8.4 (4.04–28.84)	8.4 (4.04–28.84)	*P* = 0.19
Thrombosis at diagnosis (*n* (%)):	0	13 (4%)	13 (3.4%)	*P* = 0.61
Arterial		13 (4%)	13 (3.4%)	
Venous		0	0	
Thrombosis during follow-up (*n* (%)):	6 (23%)	32 (9.9%)	38 (10.9%)	*P* = 0.08
Arterial	4 (15%)	26 (8.1%)	30 (8.6%)	*P* = 0.26
Venous	2 (8%)	6 (1.8%)	8 (2.3%)	*P* = 0.11

^a^Median (range)

We analyzed efficacy, toxicity and risk of leukemic evolution in these settings of patients. Intolerance and resistance to HU were categorized according to ELN criteria for ET^8^. Non-parametric tests, such as Mann–Whitney, Pearson Chi-square and Fischer’s exact tests, were used for statistical analysis of continuous and categorical variables. Leukemia-free-survival (LFS) curves were calculated by Kaplan–Meier method and compared with Log-rank (Mantel-Cox) test.

Twenty-six of 30 patients were evaluable for full demographic, diagnostic and therapeutic information and were considered for statistical analysis. As reported in Table 1, we observed some significant differences between BU patients and the entire series of ET patients with respect to age and blood counts at diagnosis. The BU patients were older at diagnosis and presented higher platelet (PLT) count and lower levels of hematocrit (Ht), as compared to global ET population. These demographic and laboratory features could reflect some biological differences in disease aggressiveness. Therefore, the clinical outcome was not comparable in term of survival and myelofibrosis evolution.

At the beginning of BU treatment, median age of patients was 79 years (range 59–88), median Ht value, white blood cell count (WBC) and PLT count were 39.8% (range: 30.9–50.6), 6.6 × 10^9^/L (range: 3.5–35.1) and 706 × 10^9^/L (range: 240–1343), respectively. Median time elapsed from diagnosis to BU start was 3.7 years (range: 0.2–21.2). At time of the switch to second-line therapy with BU, no patient was in CHR: 11 patients (42.3%) were intolerant and 15 (57.7%) were resistant to HU. Patients received BU for a median time of 48 months (3.7–102.2), with a median cumulative BU dose of 481 mg (80–1032).

Twenty-four of 26 (92.3%) evaluable patients obtained CHR, after a median time of 7.4 months of drug exposition (range: 3.2–37.4). The median duration of CHR was 43.2 months (range 10–81.9).

Four (15.4%) patients presented with hematological adverse events (evaluated according to the National Cancer Institute Common Terminology Criteria for Adverse Events (CTCAE), version 4.0): one patient with grade 3 anemia, one case of grade 3 thrombocytopenia and two patients with grade 2 neutropenia. 75% (3/4) of them discontinued definitively the drug, with a reversible hematological toxicity over 6 months. Only one case of non-hematological cutaneous grade 2 adverse event was documented.

At the time of analysis, 16 (61.5%) patients stopped BU: 3 for grade ≥2 hematological toxicity, 4 for disease progression, 3 for drug extra-hematological intolerance or resistance, 2 for grade ≥2 infectious complications; the remaining 4 not for drug-related raisons (principally due to personal patient’s decision). Fourteen of 16 patients (87.5%) stopped BU treatment after achievement of CHR.

With a median follow-up of 130 months (range 16–333.3) from diagnosis and 72,7 months (range 5–105.2) from BU start, 6 (23%) patients have died. Causes of death were disease progression in 3 patients (*n* = 2 leukemic evolution, *n* = 1 MF transformation), infections (*n* = 1) and unknown (*n* = 2). Thrombotic complications after BU start were observed in 5 (19.2%) patients (3 arterial and 2 venous thrombosis). All patients received anti-platelet therapy associated to BU cytoreduction, according to standard guidelines of treatment for ET^2^. No major bleedings or second neoplasms were recorded.

**Fig. 1:**
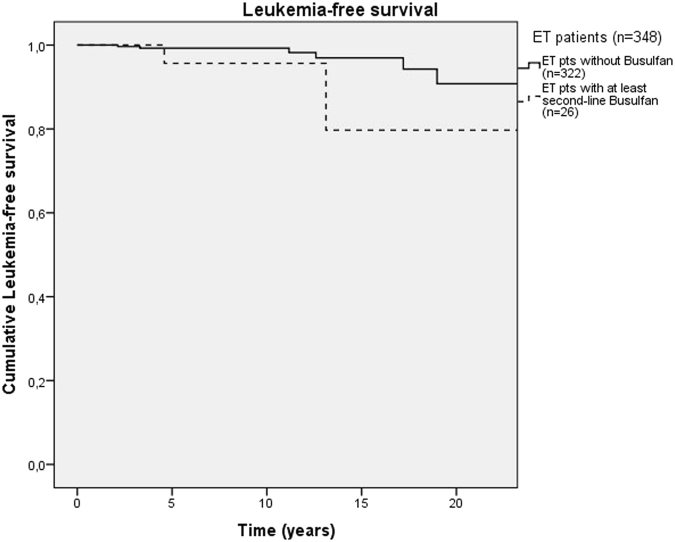
Leukemia-free survival (LFS) in patients (pts) with Essential Thrombocythemia (ET) treated with Busulfan (*n* = 26) and without Busulfan (*n* = 322)

Leukemic evolution was observed in 2 (7.7%) patients after a median time of 31.5 months from BU start and 104.8 months from diagnosis. Despite the demographic and biological differences in disease aggressiveness of BU patients, this incidence of transformation into acute leukemia was similar to the entire series of ET patients (2.6%, *P* = 0.14). The LFS at 15 years was comparable in 2 groups (97 vs. 79.7% at 15 years; Fig. 1, *P* = 0.12).

In conclusion, to our knowledge, the present study represents the first experience with an alternative long-term and low dose administration of BU in elderly patients with ET who are resistant or intolerant to HU. This different schedule seems to be safe and effective. It provides a high rate of hematological response (92.3% of patients obtained CHR) with acceptable hematological and extra-hematological toxicity. The achievement of CHR is rapid (6.5 months) and sustained over time (43.2 months). The risk of leukemic transformation seems to be limited and similar to that is reported in recent literature^4,5^, considering that these patients are very old and previously treated^3,4^. Additional data from larger retrospective multicenter studies or prospective series should be further obtained to confirm the long-term safety and efficacy of this alternative schedule of BU in ET patients.
